# Semi-Automatic Spectral Image Stitching for a Compact Hybrid Linescan Hyperspectral Camera towards Near Field Remote Monitoring of Potato Crop Leaves

**DOI:** 10.3390/s21227616

**Published:** 2021-11-16

**Authors:** Pierre Chatelain, Gilles Delmaire, Ahed Alboody, Matthieu Puigt, Gilles Roussel

**Affiliations:** Laboratoire d’Informatique, Signal et Image de la Côte d’Opale (LISIC, UR 4491), Université du Littoral Côte d’Opale (ULCO), F-62228 Calais, France; pierre.chatelain@univ-littoral.fr (P.C.); ahed.alboody@univ-littoral.fr (A.A.); matthieu.puigt@univ-littoral.fr (M.P.); gilles.roussel@univ-littoral.fr (G.R.)

**Keywords:** spatio-spectral scanning, hyperspectral stitching

## Abstract

The miniaturization of hyperspectral cameras has opened a new path to capture spectral information. One such camera, called the hybrid linescan camera, requires accurate control of its movement. Contrary to classical linescan cameras, where one line is available for every band in one shot, the latter asks for multiple shots to fill a line with multiple bands. Unfortunately, the reconstruction is corrupted by a parallax effect, which affects each band differently. In this article, we propose a two-step procedure, which first reconstructs an approximate datacube in two different ways, and second, performs a corrective warping on each band based on a multiple homography framework. The second step combines different stitching methods to perform this reconstruction. A complete synthetic and experimental comparison is performed by using geometric indicators of reference points. It appears throughout the course of our experimentation that misalignment is significantly reduced but remains non-negligible at the potato leaf scale.

## 1. Introduction

### 1.1. Context

Near in situ agriculture observation has experienced a massive boom in recent years with the arrival of the big data era. Plant disease monitoring is of fundamental interest for crop management. Several studies related to different kinds of cultures have thus emerged, e.g., corn [1], wheat [2], grapes [3], or potatoes [4].

Nowadays, hyperspectral imaging (HSI) appears as an interesting track for agriculture to measure the plant phenotype [5]. HSI provides datacubes with two spatial and one spectral dimension. Observing the spatial pixels along the spectral dimension allows to analyze the spectral content of small areas. Specifically, health plant monitoring systems are devised from multiple embedded systems, such as satellites [6], aircraft [7], unmanned aircraft vehicles (UAV) [2], ground-based vehicles [8], and laboratory systems [9].

In the meantime, UAVs have become popular and affordable, so scientific experimentation with embedded sensors has emerged. Onboard sensors have to be compact and robust and should work with limited resources (energy and processing unit), especially with small UAVs with limited carrying capacities.

Among HSI compact sensors—which may be carried by UAVs—only multishot (spatio-spectral linescan) [10] ones can yield images with a large spatial spectral and time resolution. This fine resolution enables to obtain accurate radiance images of parts of vegetation crops. This kind of sensor requires a precise movement to obtain a consistent datacube. However, uncontrolled transient movements of light UAVs embedded with compact commercial GNSS controller make it difficult to recover datacubes with a reconstruction error lower than a few centimeters [11,12]. In this context, it is attractive to obtain a portable system which repeatedly moves in a very accurate way.

Contrary to multispectral imaging (MSI), where a small set of specific appropriate wavelengths are chosen, HSI covers all wavelengths within a range with a tiny step, making it possible to monitor complex phenomena over space and time, such as plant diseases. Classical linescan cameras only account for portable imagers, which can yield the content of one spatial row. However, among compact imagers, only spatio-spectral cameras—also called hybrid linescan cameras—can provide lots of spectral bands. Contrary to classical linescan cameras, which observe a narrow area in a single row with all available wavelengths, hybrid linescan cameras inspect in one shot an extended area in several spatial rows with one wavelength each. As a result, a scanning process with an accurate linear movement is required to build a complete scene.

Our sensor is provided by IMEC and is embedded in a XIMEA compact USB3 camera MQ022HG-IM-LS150-VISNIR (xiQ series). Among all the artifacts brought by this HSI camera, the main challenging step for precision agriculture consists in image registration in order to obtain a consistent datacube. Indeed, the angle of view—which is different from the ground reflected vertical ray, also called NADIR—of different spectral bands associated with various vegetation leaf heights leads to non-rigid object movements in the scene. This effect cannot be neglected at the pixel scale, and corrective steps such as orthorectification are needed to narrow this geometric effect by referring, for example, to image stitching methods.

### 1.2. Related Works

Generally, image stitching is tackled over pairs of RGB images presenting a significant overlapping area with different viewing angles. One classical way to tackle this problem is a feature-based registration approach. It usually involves a two-step technique [13]. The first one is to look for a putative set with matching pairs of points between images, which is usually performed with the search for invariant features [14]. It is usually followed by a curative step that aims at removing false matches, also viewed as outliers. This step enables providing a clean matching set.

Image alignment, which considers the optimal matching set as the input, is often performed through a single global transformation [15], but misalignments still exist, due to model inadequacy. To overcome this problem, some authors proposed some local projective models, such as as-projective-as-possible warp [16] or shape-preserving-half-projective warp [17], which warps locally according to the proximity of the reference points.

However, these techniques cannot be applied directly to pairs of hyperspectral datacubes because each pixel has nearly two hundred bands, which cannot be realigned independently for computation reasons. Instead, a reference layer is chosen, and the extraction of coordinate feature points is performed between two identical reference layers from both datacubes. Then, reference layer stitching is performed by finding a single adequate model between the reference layer from datacube 1 and datacube 2. The same model is applied to other layers since each datacube is already aligned within layers [18].

### 1.3. Our Contribution and Paper Organization

In our case, extracting feature points directly from raw images is impossible since raw images involve several spectral bands which have their sensing specificity and sensitivity. Instead, once a first datacube reconstruction is performed, there still exists misalignments between layers. Contrary to classical image stitching or datacube stitching, a collection of warping models has to be learned with respect to a reference layer. However, searching for feature points in each layer is tedious, so extrapolating them with feature points from a few spectral layers feature appears as an interesting solution.

**Figure 1:**
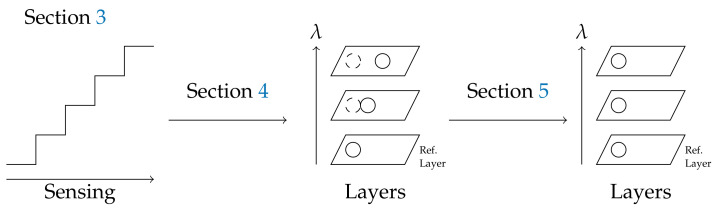
Big picture.

In this paper, the hybrid linescan camera is introduced. We propose two new approximate datacube reconstruction methods, which both assume a regular linear move of the sensor, but which highlight two different points of view. The first one is based on heuristic shifts between raw frames, while the second one uses the physics of the sensor. However, spectral layers obtained from both methods still contain misalignments, due to non-rigid object moves, according to the spectral band and object height. A reference spectral layer is chosen as the one obtained with NADIR rays, and it is considered geometrically unbiased. As a result, any spectral layer is aligned on the reference spectral layer by identifying a specific warping homography for each layer. As an illustration, the whole procedure is summarized in Figure 1. A comparative study of state-of-the art-stitching methods is performed to evaluate the quality of the corrective step.

The remainder of the paper reads as follows. Firstly, it emphasizes projective warps, which maps the matching points from one image to another. It then focuses on the specificity of our hyperspectral camera to develop an approximate spectral recovery. However, given that objects from various spectral planes are not well spatially located, various stitching methods are proposed for each spectral layer. It is then followed by practical experimentation, which shows a significant improvement of the geometric scattering of reference points.

## 2. Projective Warps

### 2.1. Inlier Set of Matching Pairs

Image alignment is often tackled between a pair of selected images. The first step to perform image alignment is to get an input set of matching points, i.e., a set of $N_{P}$ putative corresponding points described as $P≜{\left\{\left(p_{j},q_{j}\right)\right\}}_{j=1}^{N_{P}}$, where $p_{j}$ accounts for the coordinates in input Image 1, whereas $q_{j}$ accounts for the corresponding coordinate vector in Image 2. This is usually achieved by applying feature detection algorithms that look at the invariant feature descriptors in each image, such as scale invariant feature transform (SIFT) [19] or speeded up robust features (SURF) [14]. However, the putative set is usually embedded with false matches, which may corrupt the alignment step, so some specialized algorithms are devoted to removing mismatches [20,21]. In some cases, the automatic generation of an input set is not feasible for several reasons, especially in agricultural crops, where plant patterns are repetitive, so it calls for the manual extraction of reference points [12,22].

### 2.2. Global Homography Warping (GHW)

Consider that we get an optimal inlier set $I≜{\left\{\left(p_{j},q_{j}\right)\right\}}_{j=1}^{N_{I}}\subset P$—where $N_{I}$ stands for the number of feature points from this set—which describes the mapping between 2D coordinates from Image 1 to Image 2. A homography matrix *H* relates homogeneous coordinates $p_{j}\;≜\;{\left[x_{j},y_{j},1\right]}^{T}$ from image 1 to its corresponding points in the second image $q_{j}\;≜\;{\left[u_{j},v_{j},1\right]}^{T}$ which can be written as follows:

(1) $$\forall j\in \left\{1\cdots N_{I}\right\}\;q_{j}∼\left[\begin{array}{ccc} h_{1,1} & h_{1,2} & h_{1,3}\\ h_{2,1} & h_{2,2} & h_{2,3}\\ h_{3,1} & h_{3,2} & 1 \end{array}\right]\;p_{j}≜H\;p_{j}$$

where ∼ accounts for collinearity between both vectors. Equation (1) is also called global homography warping (GHW). Another point of view consists in considering two orthogonal vectors to $q_{j}$, e.g., $\left[0,-1,v_{j}\right]$ and $\left[1,0,-u_{j}\right]$, which may be used to obtain equivalent equations, i.e.,

(2) $$\forall j\in \left\{1\cdots N_{I}\right\}\;0_{2\times 1}=\left[\begin{array}{ccc} 0_{1\times 3} & -p_{j}^{T} & v_{j}\;p_{j}^{T}\\ p_{j}^{T} & 0_{1\times 3} & -u_{j}\;p_{j}^{T} \end{array}\right]\;h=A_{j}\;\cdot \;h$$

where h≜vec(H) and $A_{j}$ may be read from Equation (2). Stacking Equation (2) for all coordinates belonging to the inlier set I enables to introduce a global matrix *A* defined as $A≜{\left[A_{1}^{T}A_{2}^{T}\ldots A_{N_{I}}^{T}\right]}^{T}$ such that $A\;\cdot \;h=0_{2N_{I}\times 1}$.

Instead of exact equality, this search is usually formulated by looking for the least significant singular vector under an optimization framework, i.e.,

(3) $$\min_{h}{\;||A\;\cdot \;h||}_{F}^{2}$$

This formulation is often regarded as being very sensitive to noise, which corrupts the point coordinates from the inlier set [4,23], but some simple coordinate changes are able to improve the result quality [23].

### 2.3. Improved Warping Methods

A single homography relies on the assumption that all the objects from the scene belong to the same plane, which is scarcely true. As a consequence, local homographies are carried out by dividing the image into small grids [16] and by computing local weights according to the proximity of reference points to the grid center. This gives rise to a set of weighted optimizations which may be written as follows:

(4) $$\min_{h_{∆}}\;||\left(W_{∆}\;A\right)\;\cdot \;h_{∆}{\left|\right|}_{F}^{2}\;\;\forall \;∆\;\;\in \;\left\{1\ldots N_{∆}\right\}$$

where ∆ accounts for a grid number of the scene among $N_{∆}$ grids, and *W*_∆_ results from the computation of local weights derived from the grid center [16]. This method is denoted as as-projective-as-possible (APAP).

As an alternative, dual homography warping (DHW) [24] may be performed by dividing the matching set into two groups, each one having its own homography model. Any pixel in the scene is subject to its specific warping model resulting from a convex combination of these two homography models. Indeed, the current convex combination coefficient is computed from the ratio of distances to each cluster center, which makes the DHW model specific to the current pixel [24].

In addition, both methods consider implicitly that a scene is made of a few continuous zones, where objects from the same zone belong to the same plane. Moreover, the plane change occurs at the border of a specific region of 2D images. This assumption is sometimes difficult to check with complex scenes.

Furthermore, image stitching algorithms are not able to perform datacube stitching. Only Zhang [18] proposed a robust elastic warp that fits first to the central spectral plane from one cube to another and then adapts the transformation to other spectral planes.

Nevertheless, owing to the sensor structure, neither image stitching algorithms nor datacube stitching ones can be applied to raw images. A first datacube reconstruction is expected to perform multiple-layer stitching jobs.

In addition, even when a first datacube is available, none of the previous stitching methods are directly applicable to perform several layer stitching tasks, but adapting them seems to be a good strategy. As an illustration, Table 1 which gathers the assumptions related to the scene of each previous method together with the advantages and inconveniences is introduced. Note that our new methods in Table 1 are denoted without the term “collection of” for the rest of the article since the basic ones are not able to be applied in the context of spectral layer alignment.

## 3. Methodology

In this section, a complete description of the camera and its carrier system is performed.

### 3.1. Spatio-Spectral Scanning

**Figure 2:**
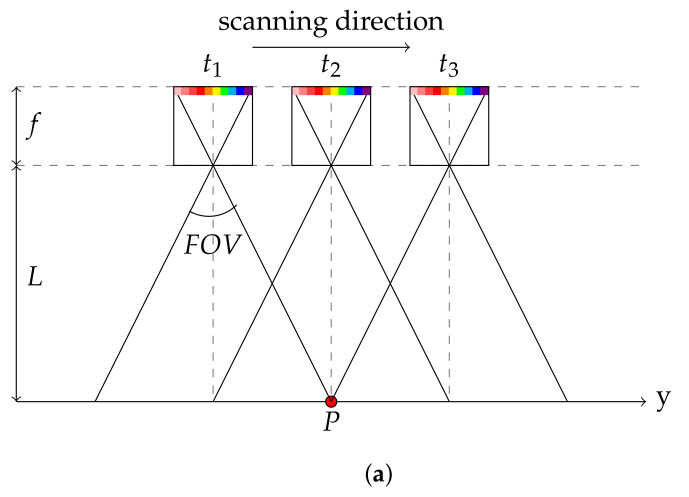

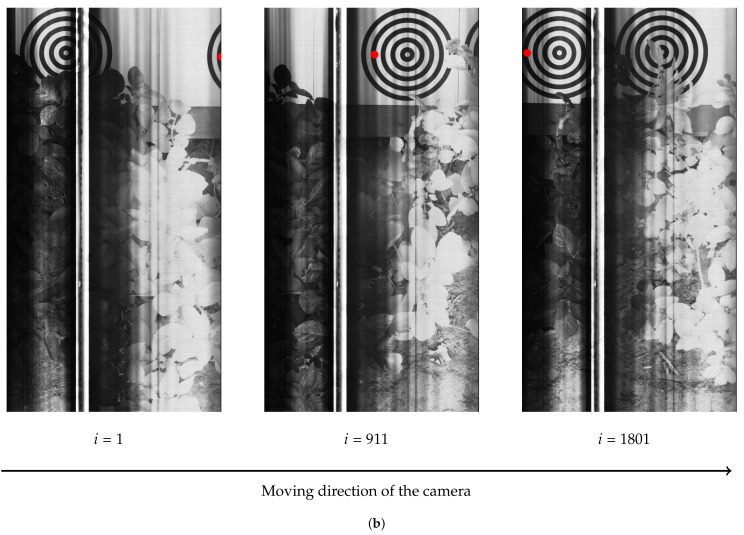
An example of camera scanning and raw data acquisition. (**a**) Schematic side view of our camera observing the scene. (**b**) Three raw images which track a point in, respectively, the extreme visible area, the NADIR area and the extreme NIR area.

Our hyperspectral camera is provided by IMEC and falls into the category of compact spatio-spectral imagers [25]. It differs from classical linescan cameras in the sense that it does not provide a complete spectral description of a spatial line in one shot. It can acquire a complete scene within a range [460;901] nm by moving linearly along the *y* direction as shown in Figure 2a since only one wavelength is obtained at one spatial position. Figure 2a illustrates that a point *P* in the scene may be observed by all the wavelengths by acquiring several images $\forall t\in [t_{1},t_{3}]$. Figure 2b shows the tracking of a feature point in both the central and extreme angles, which correspond to central and extreme band numbers.

Practically, a bank of raw images is acquired at a frame rate $f_{e}$ and it is indexed with the variable $i\in N^{*}$, which is related to the acquisition time, i.e.,

(5) $$t=t_{1}+\left(i-1\right)\times \frac{1}{f_{e}}$$

### 3.2. Sensor Structure

**Figure 3:**
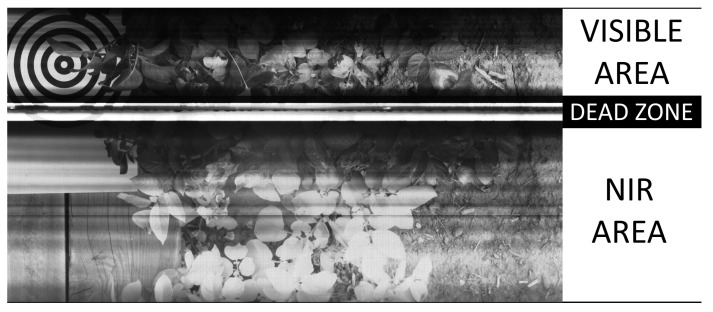
A raw image.

Raw images obtained from this sensor have a resolution of 2048 pixels width (columns) by 1088 pixels long, which are viewed as rows. Figure 3 shows a typical raw image whose description is specified below:

1. The 4 first rows are not used.
2. 64 spectral stripes (also called bandlets) of 5 by 2048 pixels each enable to inspect the visible wavelength range, represented in the top part of Figure 3.
3. A 120 by 2048 pixels rectangle (corresponding to 24 stripes) accounts for a blind area. This area corresponds to a white rectangle in the middle of Figure 3.
4. 128 spectral stripes of 5 by 2048 pixels each explore the near infrared domain (NIR).
5. The 4 last rows are not used either.

As a summary, a raw image contains 192 useful spectral bandlets covering both visible and NIR wavelengths. Each pixel from the raw image captures not only different spectral information, but also a different position in the scene.

### 3.3. 3-Axis Representation

**Figure 4:**
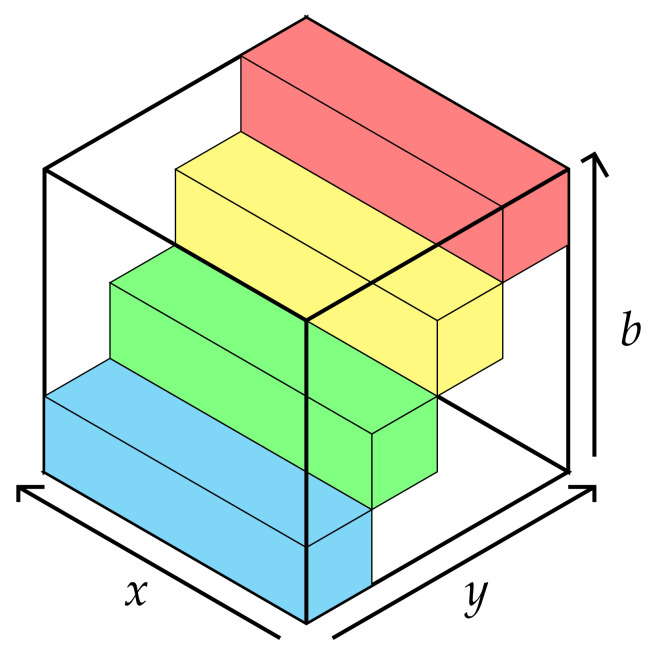
A 3D view of a raw image with 4 spectral bands.

By adding a wavelength axis as a third dimension, a raw image may be converted into a stair, as shown in Figure 4, whose step is equal to the step wavelength.

**Figure 5:**
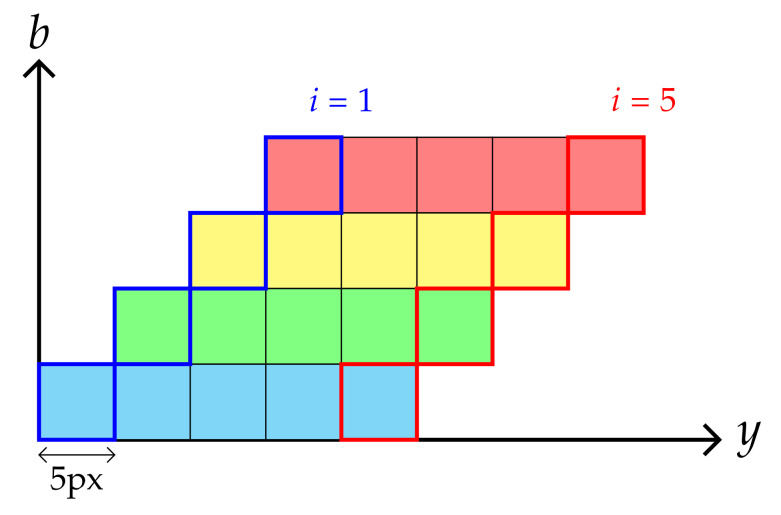
Side view of the stair stacking procedure with a 5-pixel shift.

A compact datacube may be built by stacking multiple stairs obtained from successive raw images along the scanning direction (y-axis). Owing to the stripe width (5 pixels), a displacement between two successive images strictly equal to 5 pixels corresponds to the case where successive stairs can be stacked together without any free space (Figure 5).

A shift between two successive images greater than 5 pixels leads to free space between the stairs corresponding to missing data. In cases such that the shift is lower than 5 pixels, successive stairs overlap along the y-axis, enabling to yield redundant data. This choice corresponds to the operating conditions for our study, where an interpolation task between redundant data may be carried out. However, accurate control of the shift between two successive images is not easy, except with laboratory systems. As a consequence, we chose to use such a system, which enables better control of the overlapping along the acquisition process. The description of our laboratory system is provided in the experimental section (Section 6.2).

## 4. Two Proposed Spectral Stitching Methods

We here propose two approximate spectral reconstruction methods as a first step of the datacube design. The first one is based on heuristic spectral reconstruction, while the second one uses the physics of the hybrid linescan sensor.

### 4.1. Heuristic Based Spectral Reconstruction (HSR)

**Figure 6:**
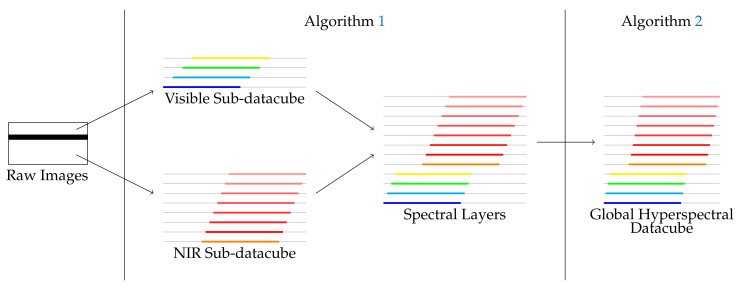
Heuristic-based strategy overview.

In this section, we propose a new spectral stitching method for hyperspectral data reconstruction. This method is composed of two steps, as described in Figure 6:

1. A sub-datacube reconstruction for hyperspectral image reconstruction and extraction of hyperspectral bands;
2. A fusion of sub-datacubes based on matching procedures.

A hyperspectral raw frame has three zones: visible, dead and NIR. The dead (blind) zone splits each frame into two zones: the first one involves 64 visible spectral bands, while the second one includes 128 near-infrared (NIR) spectral bands. To reconstruct a sub-datacube in each zone (visible and NIR), the first step is based upon finding a frame step number shift, which derives from a spatial drift between manually extracted feature points from raw images. Therefore, hyperspectral layers for visible and NIR images may be built by stacking several stairs from regularly chosen raw images. Then, it results in two independent sub-datacubes issuing from these two zones. Finally, in a second step, image matching is performed between each layer of the two sub-datacubes and a chosen reference layer.

#### 4.1.1. Sub-Datacube Reconstruction

**Figure 7:**
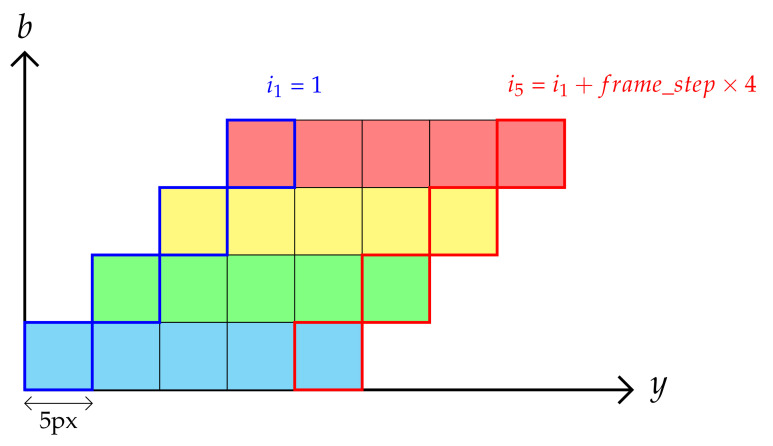
Side view of the stair stacking procedure with a frame_step pixel shift.

In this subsection, hyperspectral images are reconstructed from raw images by extracting spectral bands for visible and NIR layers. To achieve this goal, each sub-datacube is built by stacking a stair from a particular frame as described in Figure 7, and performing the same job with the frame number increased by the frame step parameter. Let $i_{n-1}$ be the current frame number, then the next frame number $i_{n}$ to be considered is expressed as follows:

(6) $$i_{n}=i_{n-1}+frame\_step$$

where *n* is the stair number. Since the index $i_{n}$ should be an integer, the frame step should also be an integer. If its estimation is not an integer, it could then be approximated as an integer.

Once the right frame number is selected, the second question is which the y-coordinate from the datacube corresponds to the selected band in the selected raw image. The answer derives from the definition of a global offset. First, from the IMEC configuration file of our hybrid linescan camera, two characteristic offsets are available from the raw image definition (Section 3.2):

(7) $$\left\{\begin{array}{cc} offset\_visible & =4\\ offset\_NIR & =offset\_visible+(64+24)\times 5=444 \end{array}\right.$$

Then, the $y_{s}$ position on the sensor of the beginning of the stripe number *b* may be deduced from the sensor structure, i.e.,

(8) $$\left\{\begin{array}{cc} y_{s} & =offset\_visible+1+(b-1)\times 5\;if\;b\in \{1\cdots 64\}\\ y_{s} & =offset\_NIR+1+(b-1)\times 5\;if\;b\in \{65\cdots 192\} \end{array}\right.$$

Then, the stripe coming from the $n^{th}$ stair for band *b* should be stored at position $y_{n}^{b}$ in the sub-datacube. Its expression is derived from Figure 7 and is expressed as follows:

(9) $$\left\{\begin{array}{c} y_{n}^{b}=\left(n-1\right)\times 5+\left(b-1\right)\times 5+1\;if\;b\in \left\{1\cdots 64\right\}\\ y_{n}^{b}=\left(n-1\right)\times 5+\left(b-65\right)\times 5+1\;if\;b\in \left\{65\cdots 192\right\} \end{array}\right.$$

The reconstruction algorithm which derives from Equations (6), (8) and (9) is provided in Algorithm 1. As a result, two independent sub-datacubes are available. However, the frame step parameter frame_step should be identified for a complete procedure.
**Algorithm 1** Sub-datacube Reconstruction**set** i **to** 1**while** i ≤ nb_frames **do**   **for** b **from** 1 **to** 64 **do**     **from** frame i **copy** the 5 rows **from** position $y_{s}$ according to Equation (8)     **paste** the 5 rows at position $y_{n}^{b}$ **into** spectral plane b using Equation (9)   **end for**   **for** b **from** 65 **to** 192 **do**     **from** frame i **copy** the 5 rows **from** position $y_{s}$ according to Equation (8)     **paste** the 5 rows at position $y_{n}^{b}$ **into** spectral plane b using Equation (9)   **end for**   **update** *i* **to** *i* + frame_step**end while**

#### 4.1.2. Estimating the Frame Step Parameter

The previous procedure needs to stitch one data stair beside another one. As a consequence, the frame step parameter needs to be identified. To this end, in each area (visible, NIR), a feature point extracted manually from several regularly spaced raw frames enables to estimate a frame step expressed in pixels per stripe width (5-pixel datacube). Tracking a feature point along the frame number enables the computation of an approximate frame step. Let $i≜i^{\prime }-i$ be the frame number shift and $∆y_{s}≜y_{s}\left(i^{\prime }\right)-y_{s}\left(i\right)$, then the frame step which enables to move from one stripe to another reads as follows:

(10) $$frame\_step=\frac{∆i}{∆y_{s}}\times 5$$

The frame step parameter may also be computed in the least square sense by estimating the slope parameter of the curve *i* versus $y_{s}$. Practically, four regularly spaced raw frames are used to compute the parameter.

However, visible and NIR sub-datacubes are not matched together, due to the integer rounding of the frame step parameter. In fact, a drift will occur along with the datacube design. Indeed, if the real value of the frame step parameter is, for example, 9.5 frames per band, its integer rounding may lead to an approximate value of 9. A greater number of bands is inserted in the whole datacube, which leads to a datacube stretching effect, where the latter is increasing with the band number. On the contrary, a lower number of bands is stacked if the frame step is overestimated, which corresponds to a contraction effect. As a consequence, image matching should be achieved.

#### 4.1.3. Matching and Fusion of Sub-Datacube

At this stage, the two sub-datacubes are not aligned; moreover, there are still misalignments between bands within the sub-datacube. As a consequence, the procedure proposed in this section consists of matching and fusing both visible and NIR spectral layers into a single global hyperspectral datacube.

To this aim, it can be noticed first that only part of the scene is shared by the two sub-datacubes, so unshared parts may be removed to ensure that sub-datacubes can be fused. Then, the desired datacube is built by stacking the spectral layers of each sub-datacube at the right position.

**Figure 8:**
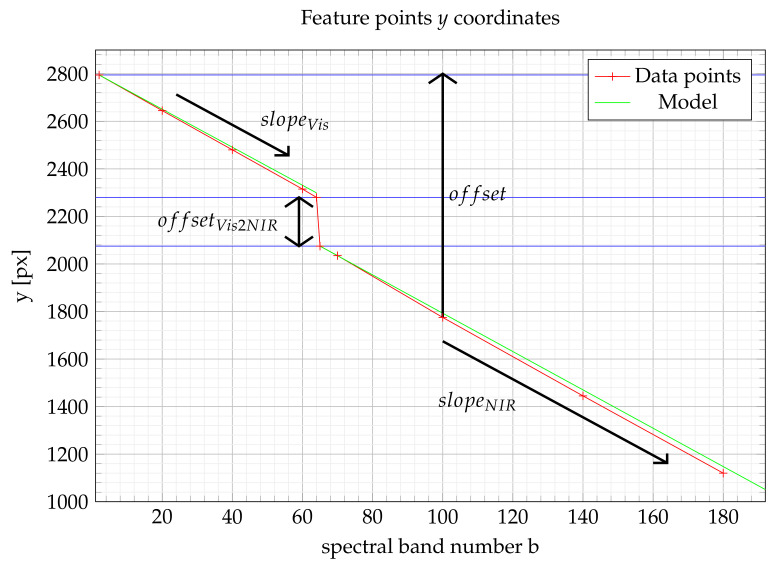
Evolution of feature point coordinates according to spectral band number.

Figure 8 describes an example of a drift of a feature point coordinate along the spectral band number *b*. So, considering that the first band is the reference, the feature point should obtain the same position in every band. As a result, the offset in Figure 8 describes the shift to be performed to reach the target position (2800 px) for every layer.

To perform this correction automatically, the target layer number *b* should be shifted according to a specific model. We propose to fit an affine model for each spectral area and then connect each model with an offset at the border of each spectral area. To that goal, a slope parameter is used for each zone (resp. $slope_{Vis}$ for visible area and $slope_{NIR}$ for NIR area). The slope parameter is calculated for each zone in the least square framework and approximated to an integer value. Then, an offset integer parameter, denoted as $offset_{Vis2NIR}$, is deduced from the difference between the border of each area.

The first spectral band is considered the reference one in this case. Figure 8 shows the shift to be performed to stitch any layer with the reference one. This shift, denoted as offset, is computed with the slope parameter of each zone and potentially the offset parameter $offset_{Vis2NIR}$. Algorithm 2 describes the new datacube obtained with each layer shifted by a computed offset, i.e.,

(11) $$\left\{\begin{array}{ccc} offset & =\left(b-1\right)\times slope_{Vis} & if\;b\in \{1\cdots 64\}\\ offset & =63\times slope_{Vis}+offset_{Vis2NIR}+\left(b-64\right)\times slope_{NIR} & if\;b\in \{65\cdots 192\} \end{array}\right.$$

This method enables to design a datacube which may be evaluated afterwards.

However, the method represented by Algorithms 1 and 2 requires the parameters to be integers. This condition implies that the frame step from Algorithm 1 is an integer. This assumption which may only be approximately checked may lead to a possible drift in the datacube reconstruction. The same drift may be observed in Algorithm 2 according to band number *b* due to an integer rounding error.
**Algorithm 2** Matching and fusion of sub-datacube.create a datacube with zero’s values**from** spectral plane 1 **copy** all**paste** all at position 1 **on** datacube(,,1)**set** offset1to$\;slope_{Vis}$**set** offsetto offset1**for** band *b* **from** 2 **to** 64 **do**   **from** spectral plane *b* **copy** all   **paste** all at position (offset,..., *b*) **on** datacube   **update** offsetto offset + offset1**end for****set** offset2to$\;offset_{Vis2NIR}$**update** offsetto offset +offset2**set** offset3to$\;slope_{NIR}$**for** band *b* **from** 65 **to** 192 **do**   **from** spectral plane *b* **copy** all   **paste** all at position (offset,..., *b*) **on** datacube   **update**  offsetto offset + offset3**end for**

### 4.2. Physical-Based Spectral Reconstruction (PSR)

**Figure 9:**
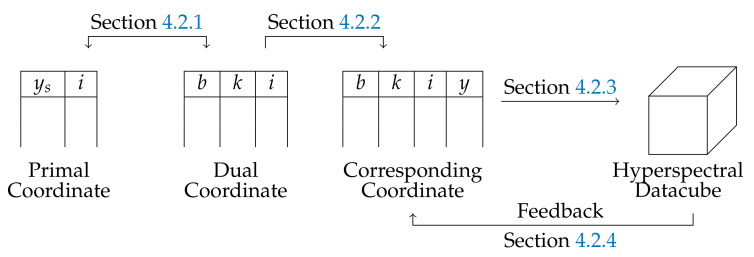
Physical-based strategy overview.

This new method takes into account the physics of image acquisition such that only one parameter is required to perform an approximate datacube reconstruction. Contrary to the previous method, this method is devoted to real parameter computation even if, in essence, indexes are integer numbers. In addition, it tries to exploit radiance redundancy in order to obtain reliable information. Figure 9 describes the different steps of the new method.

#### 4.2.1. First Basis Change

**Figure 10:**
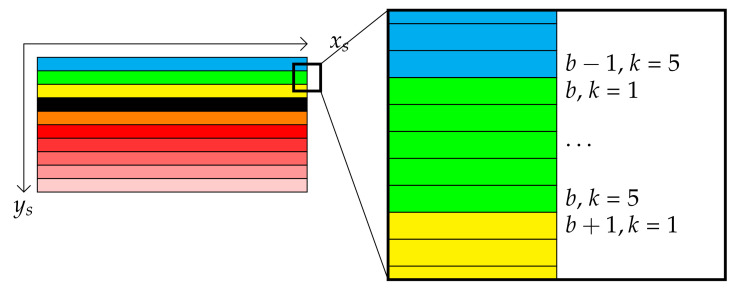
A zoom on the coordinate change.

Originally, each pixel coordinate is mapped to a single position in the coordinate system $\left(x_{s},y_{s},i\right)$, where $\left(x_{s},y_{s}\right)$ is the pixel position on the sensor for frame number *i*. Now, we would like to replace $y_{s}$ with a dual coordinate $\left(b^{*},k\right)$, where $b^{*}$ accounts for the band number ($1\leq b^{*}\leq 216$), and *k* is the shift index within a band (1≤k≤5). Figure 10 shows these dual coordinates on successive rows from any raw image.

The associated starting position for extended band number $b^{*}$ on the sensor, denoted $y_{s}$, derives from the definition of the sensor, i.e.,

(12) $$y_{s}=4+\left(b^{*}-1\right)\times 5+k$$

where $b^{*}\in \left\{1\cdots 216\right\}$ (Note that only 192 bands are really used since band 65 to 88 correspond to the dead zone of the sensor. $b^{*}$ is related to *b*, i.e., $b^{*}=b+24\times 1_{b>64}$).

Now, each pixel in the original system coordinate can be mapped to a single position in the new coordinate system $\left(x_{s},b^{*},k,i\right)$ by noticing that Equation (12) stands for integer division of $y_{s}-5$ by 5, i.e.,

(13) $$y_{s}-5=\left(b^{*}-1\right)\times 5+k-1$$

As a result, $b^{*}$ and *k* can be obtained from the integer division $y_{s}-5$ by 5, i.e.,

(14) $$\left\{\begin{array}{cc} b^{*} & =\left\lfloor \frac{y_{s}-5}{5}\right\rfloor +1\\ k & =1+(y_{s}-5)\;mod\;5 \end{array}\right.$$

where mod stands for modulo. Equation (14) enables to compute the new coordinates from the input system coordinate.

#### 4.2.2. Second Basis Change

To build a hyperspectral datacube, it is required to fill a 3D array indexed by (x,y,b) coordinates, where (x,y) account for ground axes and *b* is the true band number. So, in other words, we want to find a function that maps each pixel from each raw image in the 3D array. The sensor is assumed to move in the *y*-direction at a constant speed expressed in pixels per frame, denoted as step. This assumption leads to the new coordinate system, where it can be noticed that *y* takes into account the linear kinetic model of the sensor over time, i.e.,

(15) $$\left\{\begin{array}{ccc} x & =x_{s} & \\ y & =\left(b^{*}-1\right)\times 5+k+step\times \left(i-1\right) & if\;b^{*}\;\in \left\{1\cdots 64\right\}⋃\left\{89\cdots 216\right\}\\ b & =b^{*}-24\times 1_{b^{*}>88} & if\;b^{*}\;\in \left\{1\cdots 64\right\}⋃\left\{89\cdots 216\right\} \end{array}\right.$$

Note that some unused bands, corresponding to $65\leq b^{*}\leq 88$, enable to skip the computation of the corresponding coordinates.

#### 4.2.3. Interpolation of the Radiance

In the case where the speed is sufficiently low, the same point from the scene may be observed by several raw images in the same band $b^{*}$. This situation enables to exploit the redundancy of the scene for each band due to low-speed conditions. This may be performed using an interpolation task that enables to fill the datacube with the estimated radiance.

Let $\left(b^{*},k,i\right)$ and $\left(b^{*},k^{\prime },i^{\prime }\right)$ be the coordinates observing the same point, then it leads to the following equation by equating *y* with both parameters as follows:

(16) $$k+step\times \left(i-1\right)=k^{\prime }+step\times \left(i^{\prime }-1\right)$$

As a result, a relationship between these parameters should be checked, i.e.,

(17) $$k^{\prime }-k=step\times \left(i-i^{\prime }\right)$$

However, this relationship is scarcely exact since *i* and *k* are both integers while the step parameter is not. Instead, it is usually searched for a set of indexes (i,k) which approximates every target integer coordinate *y* for band number *b*, i.e.,

(18) $$S_{y}^{b^{*}}=\{\left(i,\;k\right),\;s.t.\;|y-\left(b^{*}-1\right)\times 5+k+step\times \left(i-1\right)\left|\leq 1\right\}$$

This set $S_{y}^{b^{*}}$ gathers all pairs of candidates for a fixed value of both the target coordinate *y* and band number $b^{*}$. In practice, these candidates from this set are looking at the same object in the scene. As a result, they may be used to perform an interpolation job.

Let us suppose for the sake of clarity that *y* from Equation (15), denoted as $y_{k,i}^{b^{*}}$, corresponds to the coordinate with band number $b^{*}$, shift index *k*, and image number *i*.

A weighted interpolation of radiance values may be performed according to the distance $|y-y_{k,i}^{b^{*}}|$. For this purpose, we introduce the weight parameter for every member of the set $S_{y}^{b^{*}}$ as follows:

(19) $$p_{k,i}^{b^{*}}=\frac{\left(1-|y-y_{k,i}^{b^{*}}|\right)}{\sum_{\left(i,k\right)\in S_{y}^{b^{*}}}^{}\;(1-|y-y_{k,i}^{b^{*}}\left|\right)}$$

A weighted average of the radiance values among candidates may be carried out using the previous weight definition Equation (19), i.e.,

(20) $${\hat{I}}_{y}^{b^{*}}=\sum_{\left(i,k\right)\in S_{y}^{b^{*}}}^{}\;p_{k,i}^{b^{*}}\;I_{k,i}^{b^{*}}$$

where $I_{k,i}^{b^{*}}$ accounts for the vector of radiance for a complete row (with *x* fixed, corresponding to 2048 pixel values) obtained with all pairs of indexes $\left(i,k\right)\;\in \;S_{y}^{b^{*}}$ while ${\hat{I}}_{y}^{b^{*}}$ stands for the estimated radiance row vector at coordinate *y* for band number $b^{*}$.

Algorithm 3 summarizes the different steps explained before.
**Algorithm 3** Physical-based spectral reconstruction (PSR).Create a datacube with zero’s values and size (nb_lines, nb_columns, nb_bands)**for** band $b^{*}$ **from** 1 **to** nb_bands **do**   create table of estimated position according to Equation (15)   **for** line **from** 1 **to** nb_lines **do**     According to Equation (18), search and copy all lines to create an interpolated line     according to Equations (19) and (20)     **paste** the interpolated line at position (line,..., band) **on** datacube   **end for****end for**

This formulation assumes there is no drift in the *x*-direction according to time. This hypothesis enables to perform a complete estimation of a row image. In practice, this assumption is difficult to check, and there is usually a small shift in the *x*-direction also. However, this drift is negligible for the candidates belonging to the same set $S_{y,b}$, so Equation (20) remains a reliable estimator.

#### 4.2.4. Estimating the Step Parameter

The step parameter plays an important role in the stitching task.  

**Figure 11:**
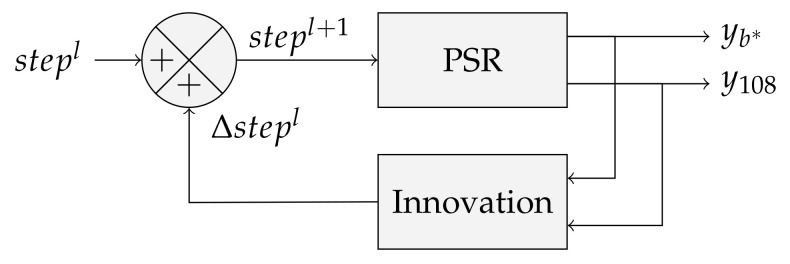
Update rule.

1. Initialization.
  The knowledge of the sensor speed enables to propose an approximate step value, expressed in pixels per frame, i.e.,
  (21) $$step^{0}=\frac{V_{d}}{\left(f_{e}\times GIFOV\right)}$$
  where $V_{d}$ [mm/s] accounts for the sensor speed and GIFOV [mm/px] stands for the ground instantaneous field of view derived from the global ground field of view [26]. Since all the parameters are known, it is easy to propose an approximate step parameter as an initial value.  
2. Update rule.
  The first run with an estimated speed parameter denoted as $\hat{step}$ leads to a first spectral reconstruction. Tracking a reference point in different bands enables inspecting a potential drift. Let $\hat{y^{108}}$ (This band corresponds to a ray angle approximately equal to 0 degrees) and $\hat{y^{b^{*}}}$ be the y-coordinate of the point from, respectively, the reference spectral band and the $b^{*}$th spectral band. Then, it turns out from Equation (15) that they may be written as follows:
  (22) $$\left\{\begin{array}{cc} \hat{y^{108}} & =k+\left(108-1\right)\times 5+\left(i-1\right)\times \hat{step}\\ \hat{y^{b^{*}}} & =k^{\prime }+(b^{*}-1)\times 5+\left(i^{\prime }-1\right)\times \hat{step} \end{array}\right.$$
  Additionally, a single coordinate of the reference point, denoted as $y_{true}$, should be expected with the true parameter step in different spectral bands so that the following holds:
  (23) $$\left\{\begin{array}{cc} y_{true} & =k+(108-1)\times 5+(i-1)\times step\\ y_{true} & =k^{\prime }+\left(b^{*}-1\right)\times 5+\left(i^{\prime }-1\right)\times step \end{array}\right.$$
  By subtracting Equation (22) with Equation (23), it results in the following:
  (24) $$\left\{\begin{array}{cc} \hat{y^{108}}-y_{true} & =\left(i-1\right)\times \left(\hat{step}-step\right)\\ \hat{y^{b^{*}}}-y_{true} & =\left(i^{\prime }-1\right)\times \left(\hat{step}-step\right) \end{array}\right.$$
  By subtracting both equations in Equation (24), an estimation of the deviation from the true value of the step parameter may be found, i.e.,
  (25) $$∆step=\left(\hat{step}-step\right)=\frac{\hat{y^{b^{*}}}-\hat{y^{108}}}{i^{\prime }-i}$$
  In order to propose a new step value, Equation (25) may be applied once to find the step increment with a single spectral band $b^{*}$ or in the least squares sense with multiple target bands to fit the best parameter. This new step value may be computed as follows:
  (26) $$step^{1}=step^{0}-\frac{\hat{y^{b^{*}}}-\hat{y^{108}}}{i^{\prime }-i}$$
  Algorithm (PSR) may then be applied with the new step parameter to obtain better stitching performance. Figure 11 illustrates a more general recursive schema, where $step^{l}$ accounts for the innovation at iteration *l* obtained from Equation (25).

Now, a first datacube reconstruction is available. In conclusion, both PSR and HSR have advantages and inconveniences. HSR is faster but less accurate in the geometric object definition, while PSR is more accurate but requires more computation time. However, both methods still need the datacube to be realigned.

## 5. Corrective Warping of the Datacube

**Figure 12:**
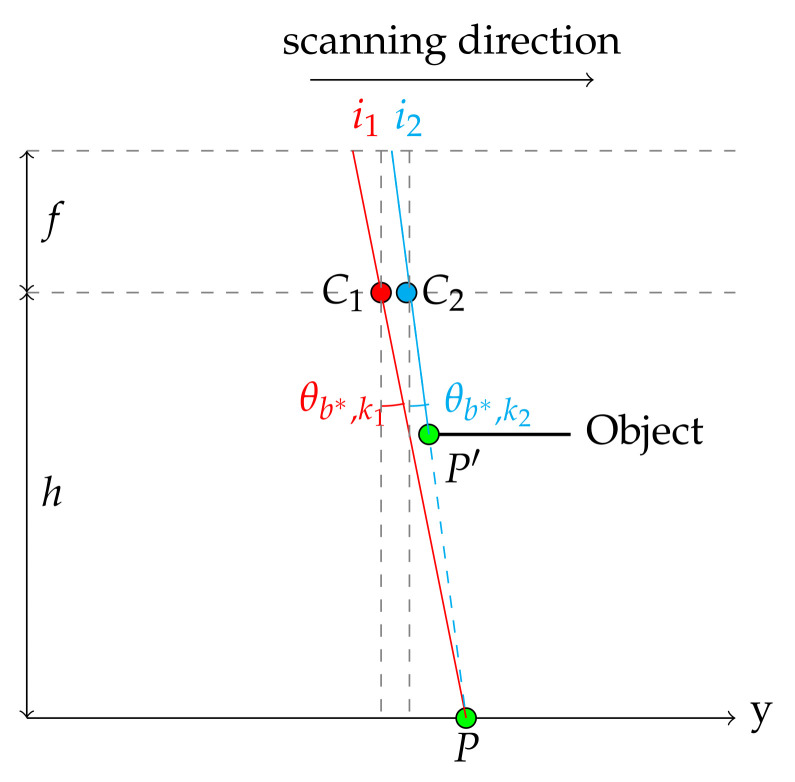
Schematic side view of 2 rays from the same band.

Once an approximate reconstruction of the datacube is performed, either with HSR or PSR, a corrective task may be considered. As accurate as the step parameter estimate may be, a physical error could persist. In addition, scanning the objects with various heights causes abrupt jumps in the y-coordinates, related to the ray angle. Figure 12 illustrates a jump in the y-coordinate due to object height variation. So, the spectral layers are still not aligned as expected.

To further improve the reconstruction, several warping methods are applied to realign the spectral layers.

### 5.1. Building the Set of Matching Pairs

As classical automatic methods (e.g., SIFT or SURF) fail in building a set of matching pairs, it is necessary to manually extract the feature points. After noticing that paired points are aligned and regularly spaced, the following two-step strategy can be used:

1. Identify several feature points in a few regularly spaced spectral layers.
2. Predict the position of feature points in each intermediate spectral layer with linear interpolation.

This step provides a set S of feature point spatial coordinates in every spectral band. The set S is defined as follows:

(27) $$S≜\{\left(x_{j}^{1},y_{j}^{1},x_{j}^{2},y_{j}^{2},\cdots ,x_{j}^{192},y_{j}^{192}\right)\;j\in \left\{1\cdots card\left(S\right)\right\}\;\}$$

This set is randomly divided into two subsets: the first one, $S_{tr}$ for the training phase, the second one $S_{te}$ for the test stage.

### 5.2. Fitting the Warping Model

First, it is important to choose a spectral layer as a reference layer. Usually, the chosen layer is the NADIR one because it is not affected by the parallax effect. It here corresponds to band number *b* equal to 84. The warping model function $w^{b\to ref}$, parameterized by the reference layer and the target layer *b*, takes as input any pixel coordinates issuing from layer *b* and provides the estimated coordinates in the reference layer, i.e.,   

(28) $$\begin{array}{ccc} w^{b\to ref}:\; & X^{b}\subset N^{2}\; & \to \;X^{ref}\subset N^{2}\\ \; & (x^{b},y^{b})\; & \to \;(x^{ref},y^{ref}) \end{array}$$

The function $w^{b\to ref}\left(.,.\right)$ transforms each pixel position from spectral layer *b* into a reference layer pixel position. According to the chosen model from Section 2, the parameters of the function $w^{b\to ref}$ are fitted for each band number *b* on the corresponding training set $S_{tr}^{b}$ extracted from $S_{tr}$ and defined as $S_{tr}^{b}≜\left\{\left(x_{j}^{b}\;y_{j}^{b}\right):\;j\in \left\{1\;\cdots card\left(S_{tr}\right)\right\}\;\right\}$. The learning phase tries to find parameters of a specific model $\eta^{b}$ which minimize the following distance for each *b*, i.e.,

(29) $$\forall b\in \left\{1\cdots 192\right\},\;\eta^{b}=\underset{\mathrm{parameters}\mathrm{of}w^{b\to ref}}{\mathrm{argmin}}\;\sum_{j\in S_{tr}^{b}}\;||w^{b\to ref}\left(x_{j}^{b},y_{j}^{b}\right)-\left(x_{j}^{ref},y_{j}^{ref}\right){\left|\right|}_{F}^{2}$$

These parameters depend on the chosen reference warping method. For the choice of a specific model, please refer to Section 2 which details different possible models.

Finally, (192−1) warping models are obtained via Algorithm 4: one for each spectral band different from the reference one.
**Algorithm 4** A global scheme to enhance and evaluate the accuracy of the reconstruction.**for** band *b* **from** 1 **to** nb_bands **do**   Fit the warping model $w^{b\to ref}\left(.,.\right)$**from** training set $\left(S_{tr}^{b},S_{tr}^{ref}\right)$   Apply $w^{b\to ref}\left(.,.\right)$ on the training set to assess the fit error between $w^{b\to ref}\left(x^{b},y^{b}\right)$   and $\left(x^{ref},y^{ref}\right)$   Apply $w^{b\to ref}\left(.,.\right)$ on the test set to evaluate the test error**end for**

### 5.3. Applying the Model and Post-Processing

Applying a warping model $w^{b\to ref}\left(.,.\right)$ consists of creating a destination layer $I^{\prime b}$ from an original layer $I^{b}$, which should, in essence, be geometrically superposed to $I^{ref}$. The intensity in $I^{\prime b}$ derives from $I^{b}$, i.e.,

(30) $$I^{\prime b}\left(w^{b\to ref}\left(x^{b},y^{b}\right)\right)=I^{b}\left(x^{b},y^{b}\right)$$

**Figure 13:**
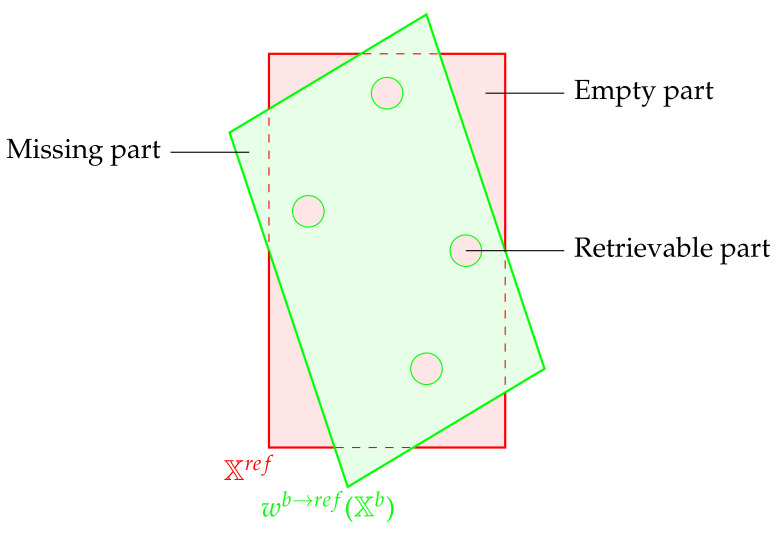
Warping issues.

In theory, the function $w^{b\to ref}$ should be bijective, so each pixel from the destination layer should be filled with the initial layer. However, in practice, the created enhanced layer may contain some of the following problems:

1. There may be some empty pixels since the warping model may not be surjective, i.e.,
  (31) $$\exists \;\left(x^{\prime ref},y^{\prime ref}\right)\in \;X^{ref}\mathrm{such}\;\mathrm{that}\forall \;\left(x^{b},y^{b}\right)\;\in \;X^{b},\;w^{b\to ref}\left(x^{b},y^{b}\right)\neq \left(x^{\prime ref},y^{\prime ref}\right)$$
  The empty pixels which are surrounded by filled pixels may be spatially interpolated by a post-processing step, as shown in Figure 13. The others are left empty.
2. The model may not be injective since two points from the original space point toward the same destination. This property translates mathematically into the following:
  (32) $$\exists \;\left(x^{\prime ref},y^{\prime ref}\right)\;\in \;X^{ref}\;\mathrm{such}\;\mathrm{that}w^{b\to ref}\left(x^{b},y^{b}\right)=w^{b\to ref}\left(x^{\prime b},y^{\prime b}\right)=\left(x^{\prime ref},y^{\prime ref}\right)\Rightarrow \left(x^{b},y^{b}\right)\neq \left(x^{\prime b},y^{\prime b}\right)$$
3. A point from $X^{b}$ may point outside from $X^{ref}$. It is represented in Figure 13 as a missing part. In such a case, the corresponding point is not taken into account.

## 6. Practical Experimentation

Our methods explained in Section 4 are applied first on a synthetic dataset, then on three experimental datasets. A comparative study of our reconstruction followed by some state-of-the-art warping methods, as described in Section 2 are performed on each dataset.

### 6.1. Synthetic Dataset

**Figure 14:**
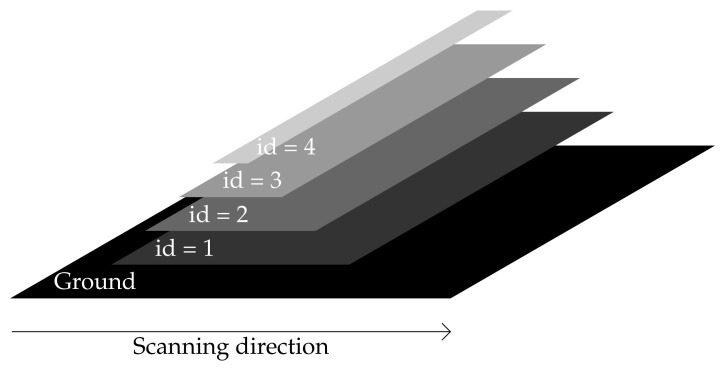
Synthetic scene.

This dataset is generated by an algorithm which simulates the reflection of light rays on the objects. As a consequence, it highlights the impact of different ray angles on the image. To this aim, the scene is composed of a stack of four rectangular planes located in specific positions described in Table 2 as shown in a perspective view in Figure 14. The background outside of these objects is taken as a black pattern.

The camera has a focal length of 12 mm. Raw images are taken every 0.1 s from a fixed altitude of 2500 mm. The ground IFOV (GIFOV) is equal in this case to 1.13 mm. The camera follows a uniform rectilinear movement with a speed equal to 11.50 mm/s so that the step parameter is approximately equal to 1 pixel per frame.

### 6.2. Real Dataset

**Figure 15:**
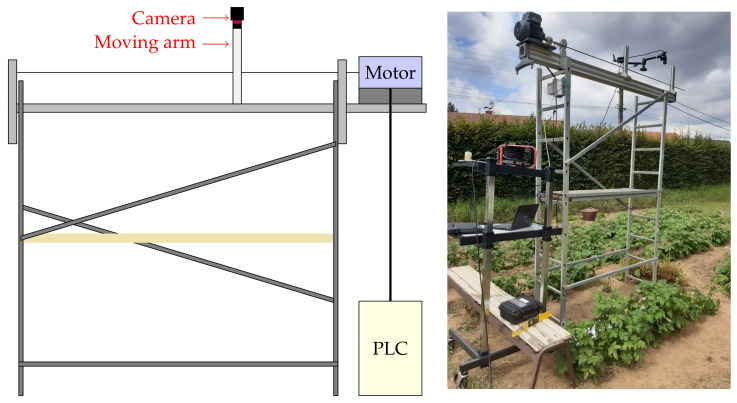
Our controlled system.

Contrary to [10], where the weather conditions may vary throughout the acquisition process, we select experimentation for which sunny and stable light conditions are guaranteed. Our laboratory system is composed of a camera carried by a moving arm which is coupled to the moving part of a linear axis. A PLC controller enables to accurately control the position of the arm to a predefined setpoint. This setpoint is chosen with a fixed speed throughout the experimentation. This system is fixed over a movable scaffolding. Figure 15 shows the current setup of our laboratory system in a private garden. Note that the speed of the camera is chosen to ensure a step parameter lower than 5 pixels per frame, which yields conditions of band overlapping.

The data were collected in Mametz, Northern France, on an outdoor potato garden. Three experiments were conducted respectively on 12 August 2020, 7 June 2021, and 19 July 2021, respectively called Mametz 1, Mametz 2, and Mametz 3. The parameters of these experiments are summarized in Table 3. It gathers several parameters, including the camera parameters and kinematic ones.

In the case of Mametz 1, the camera, driven by an open-loop laboratory system, follows an approximate rectilinear movement. The camera speed may vary within reasonable proportions, according to mechanical friction but the average speed is roughly equal to 2.6 mm/s. Contrary to Mametz 1, the camera is driven by a speed-controlled system for other experiments, where the camera speed can be considered constant throughout the course of acquisition.

### 6.3. Evaluation Index

The quality of the fit may be evaluated by inspecting the root mean square error (RMSE) for each layer, refs. [16,24,27], on either the training set ($S_{tr}$) or the test set ($S_{te}$), defined as follows:

(33) $$RMSE\left(S_{t},b\right)≜\sqrt{\frac{1}{card(S_{t})}{\left(P^{b}-Q^{ref}\right)}^{T}\;\cdot \;\left(P^{b}-Q^{ref}\right)}$$

where $S_{t}$ accounts for either the training set ($S_{tr}$) or the test set ($S_{te}$). $P^{b}$ stands for the coordinates of the estimated points in band *b* while $Q^{ref}$ are the coordinates of the reference points in the reference band. This error is converted into millimeters by dividing the error expressed in pixels by the size of one pixel GIFOV. This index is used throughout the experimentation to assess the performance of each method.

### 6.4. Results

The reference band, obtained for $b^{*}=108$ or equivalently b=84, is built with NADIR rays so it has a perfect geometric reconstruction. Extreme bands are obtained with increasing angles from NADIR. As a result, the parallax effect increases in practice with $|b^{*}-108|$. The reconstruction error RMSE is then plotted throughout the experimentation, according to the extended band number $b^{*}$ (involving the dead zone).

**Figure 16:**
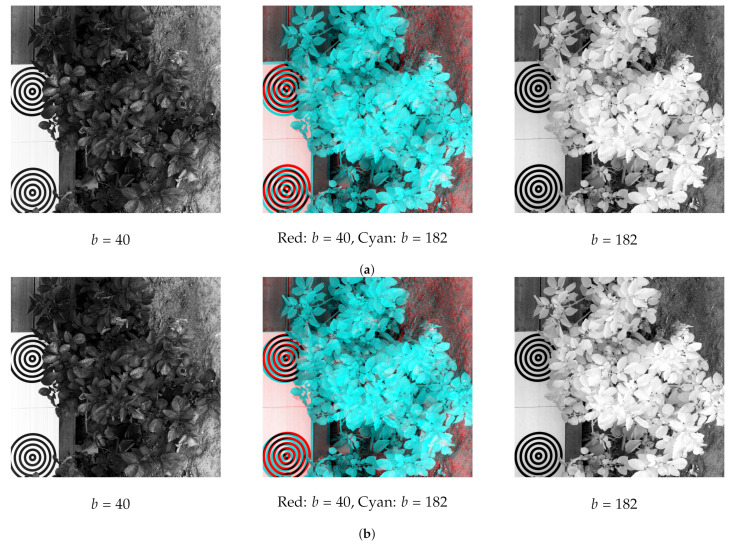
Resulting images. (**a**) HSR resulting images. (**b**) PSR resulting images.

Figure 16a,b show two resulting layers obtained from the HSR method and the PSR method, respectively. On the left and on the right part of each figure, one layer is displayed individually, while a mixture of both layers is represented in the middle. It can be noticed that both methods involve significant misalignments, which can be measured with the RMSE index.

**Figure 17:**
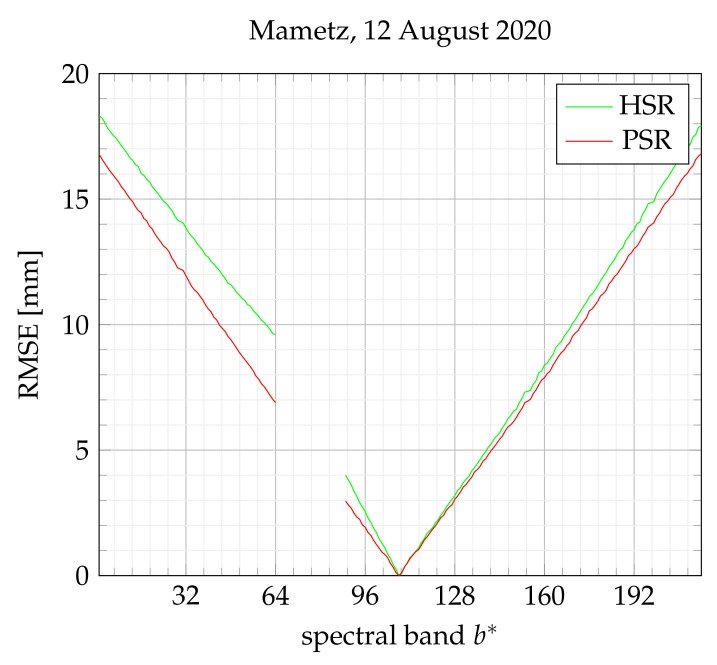
RMSE applied to approximate reconstruction (HSR and PSR).

Figure 17 intends to compare the two approximate reconstruction methods on a real dataset obtained on 12 August 2020. To achieve the comparison, the HSR datacube is resized to the PSR datacube size. As expected, the RMSE index increases with $|b^{*}-108|$. Roughly speaking, HSR and PSR appear to be equivalent. Contrary to HSR, which involves several trends in visible ($b^{*}\leq 64$) and NIR domains ($b^{*}\geq 89$), as shown in Figure 17, PSR keeps the same single trend along $b^{*}$, represented by a single slope. In our opinion, a corrective warping would be more difficult to fit on HSR, so we decided to apply all future corrective steps on PSR reconstruction only.

**Figure 18:**
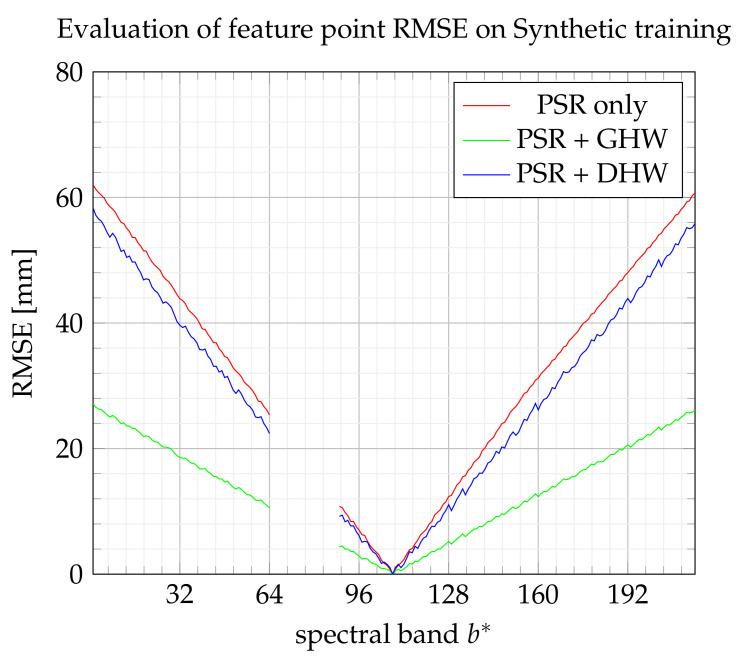
RMSE on feature points from the training set.

**Figure 19:**
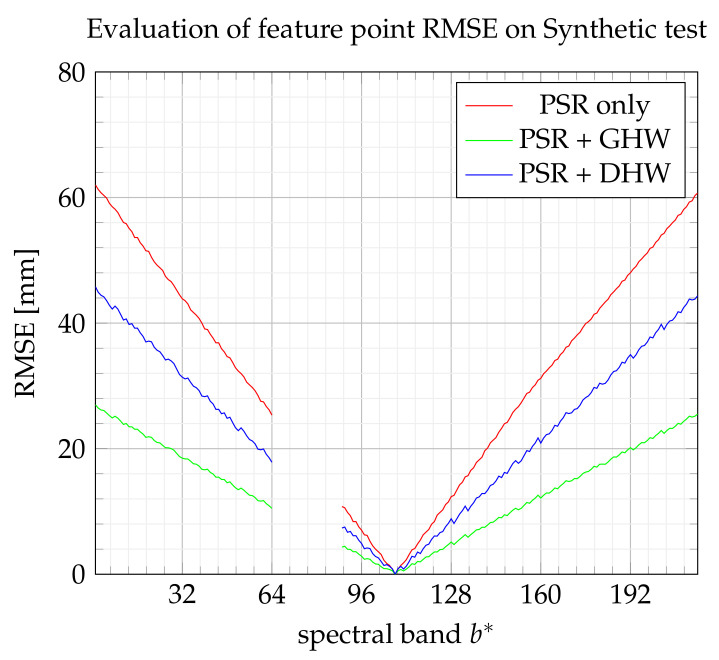
RMSE on feature points from the test set.

Figure 18 and Figure 19 show the evolution of the RMSE index according to the extended band number $b^{*}$ by referring to the training set and test set, respectively. The global homography warping (GHW) achieves the best performance. As explained in [23], without large noise on feature points, GHW often performs well, and advanced robust methods may not be useful. It may also be noticed that the improvement brought by DHW with respect to PSR appears here not significant.

**Figure 20:**
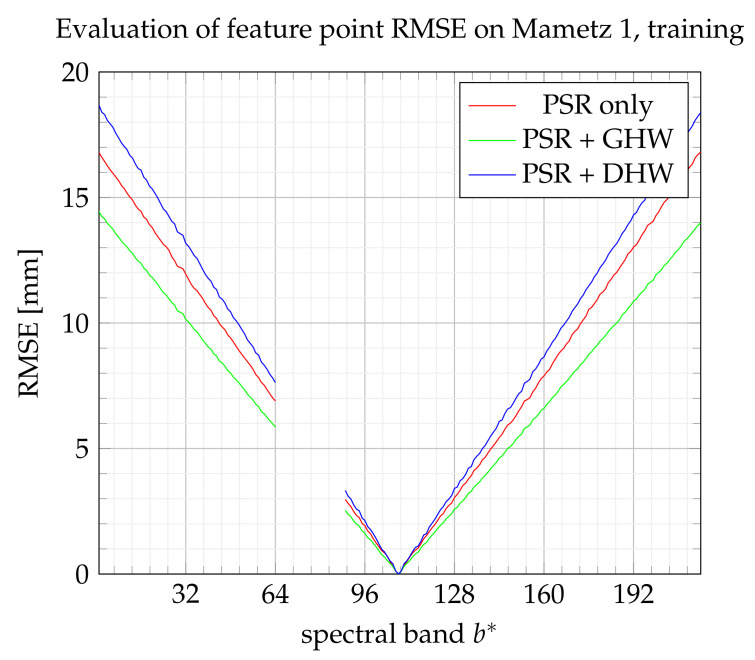
RMSE on feature points from the training set.

**Figure 21:**
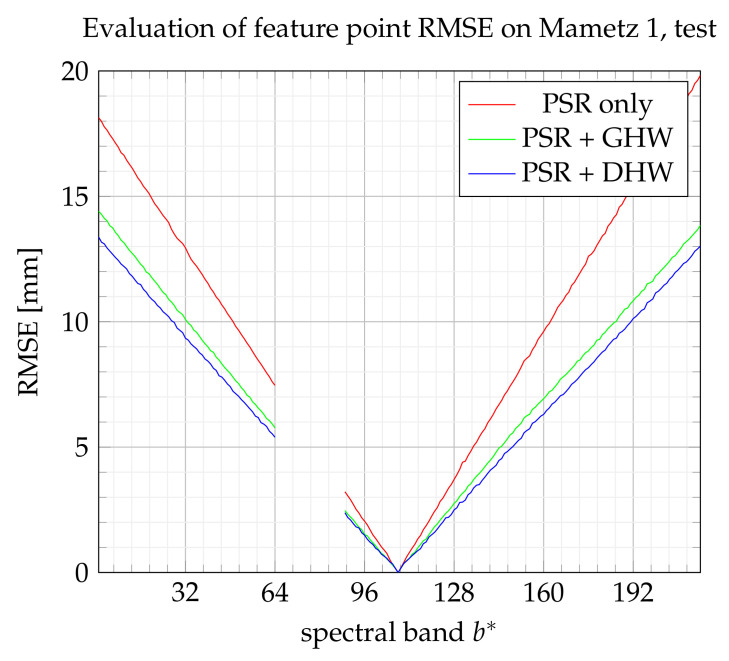
RMSE on feature points from the test set.

During the Mametz 1 experiment, fifty feature points are assigned to the training set and one hundred to the test set, which corresponds to the large sets compared with the minimum requirement for testing and training. Figure 20 and Figure 21 show the performance for the training set and the test set, respectively. Even if DHW performance looks bad on the training step, it finally appears as the best method on the test step. However, the enhancement with respect to GHW is not significant.

**Figure 22:**
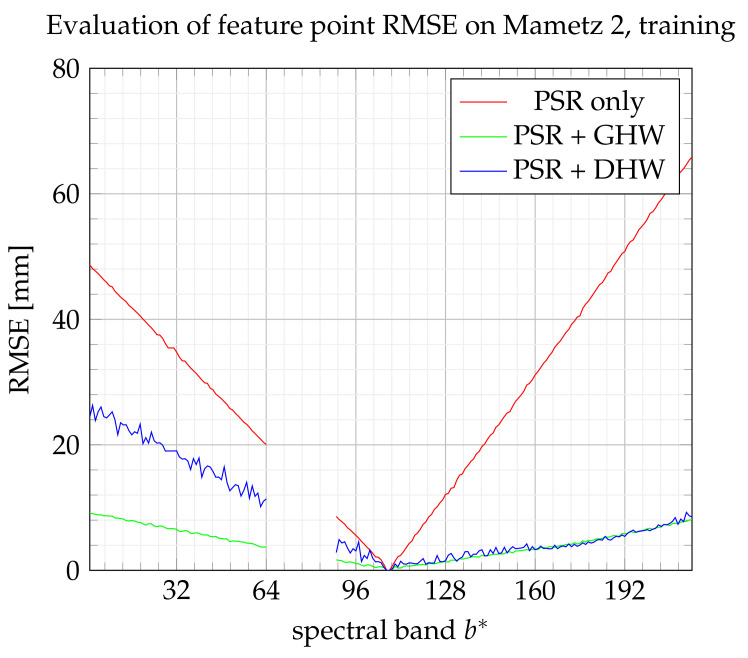
RMSE on feature points on the training set.

**Figure 23:**
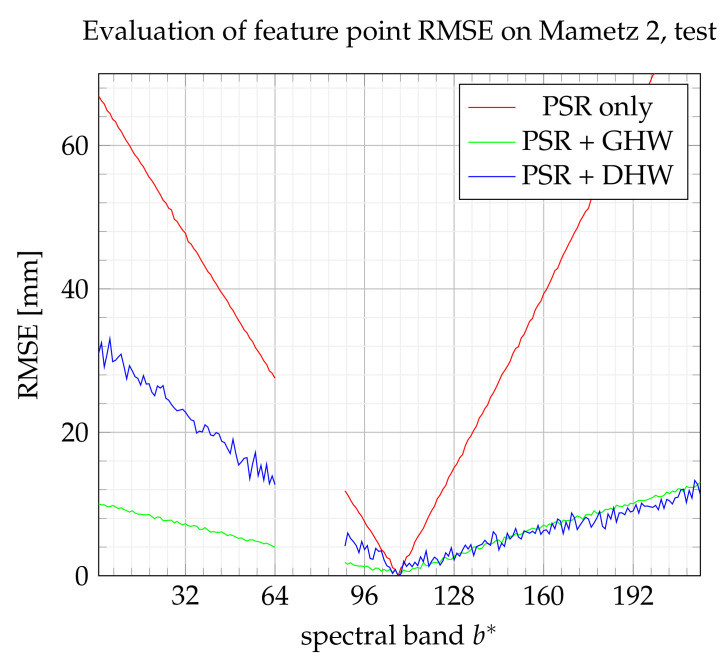
RMSE on feature points from the test set.

Figure 22 and Figure 23 show the reconstruction performance for Mametz 2. This experimentation is conducted with a small focal distance, which leads to a reduced area viewed by all bands. Indeed, at the beginning and the end of scanning experimentation, only a small number of bands observe the scene. In such a situation, a small number of feature points are registered due to the reduced common area. DHW, which divides the training set into two unbalanced subsets, involves probably only the minimum number of feature points necessary to perform the learning step. Indeed, it is observed that one feature point which is affected to one class for the NIR domain is labeled to the other class in the visible domain. As a result, the performance of DHW method decreases in the visible area, due to a mismatch of one of the two homographies. This fact causes the asymmetry of the DHW performance curve, shown in both Figure 22 and Figure 23, between the visible and NIR domains. Moreover, the noisy aspect of DHW performance may also be due to the small number of feature points from this experimentation. Globally, GHW is probably the most interesting method for its regular performance and its ease of implementation.

**Figure 24:**
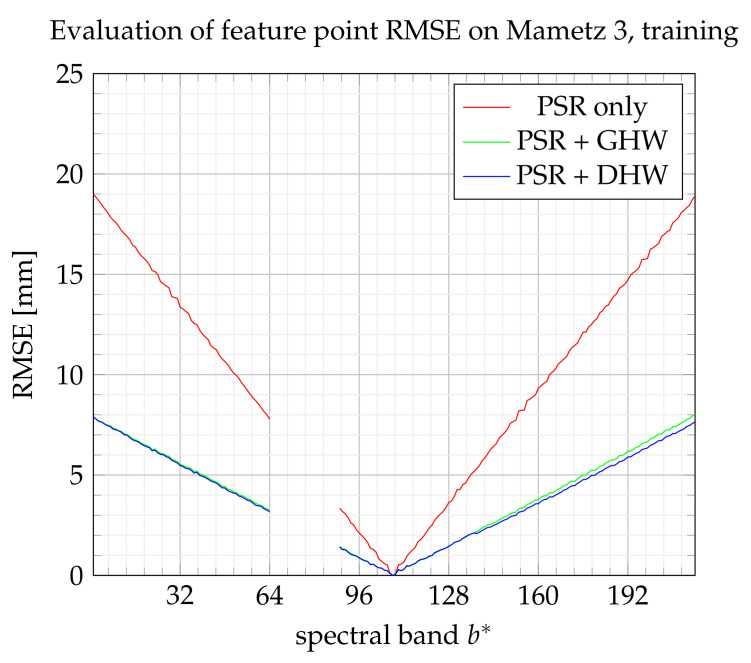
RMSE on feature points from the training set.

**Figure 25:**
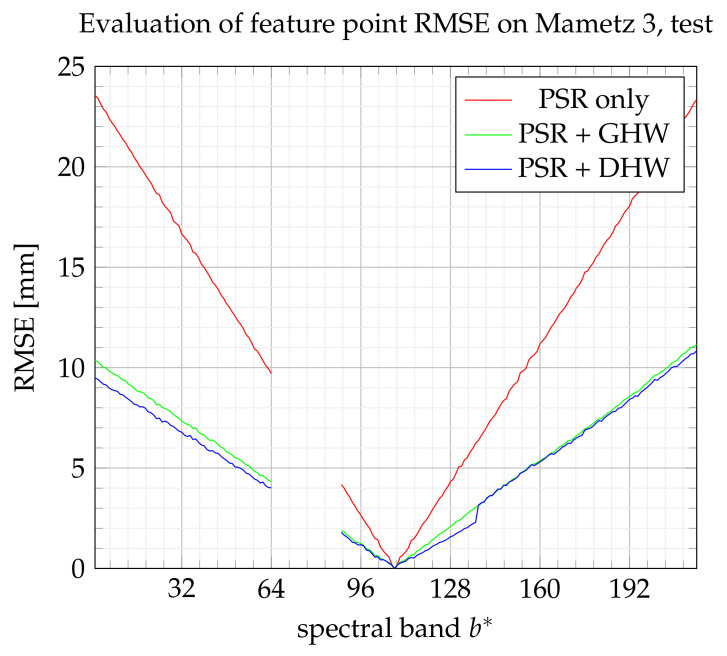
RMSE on feature points from the test set.

On Figure 24 and Figure 25, GHW and DHW turn out to be equivalent. Note also that the best performance is achieved for this experimentation among all the others, with a maximum RMSE error equal to 1 cm. Even if the RMSE could be improved, the goal in potato health monitoring is to track essentially the evolution of the red edge [28], which is performed with bands within $127<b^{*}<141$. In this range, the error remains lower than 3 mm, which is considered a reasonable drift.

To conclude, our feeling about experimentations is that using a single global homography warping (GHW) is probably preferable for further improvement. Furthermore, the most consistent experimentation is probably Mametz 3 since the camera speed is accurately controlled and the focal length is adapted for the scene observation.

Moreover, as a perspective, it should be noticed that a few homography models with an appropriate switching procedure should be considered in the future. The difficulty relies on the way the switching procedure should act.

## 7. Conclusions

In this article, we have formulated the problem of alignment of spectral layers for a specific spatio-spectral camera. After introducing the sensor structure, we defined a new coordinate system that enables to reconstruct an approximate datacube. However, the disparity of the scene object heights induces geometric misalignment, which is highly dependent on the band number. This corrective step is tackled with multiple spectral homography transformations, which enable to lower the object drifts. The reconstruction was evaluated through one synthetic dataset and three different real datasets. Though a significant improvement was achieved concerning the primary reconstruction on each dataset, the alignment accuracy still needs to be lowered regarding the size of vegetation leaves.

## Figures and Tables

**Table 1:** Comparison of previous and new method assumptions and properties.

Method	Scene Assumption	Pair of RGB Images	Pair of Spectral Datacubes	Pair of Spectral Layers
GHW [23]	Objects in the same plane	Fast	X	X
APAP [16]	Smooth changes	Slow	X	X
DHW [24]	Two distinct planes	Medium	X	X
SPHP [17]	Two distinct planes	Slow	X	X
Zhang [18]	Smooth changes	X	Fast	X
Collection of GHW (ours)	Objects in the same plane	X	X	Medium
Collection of DHW (ours)	Two distinct planes	X	X	Slow

**Table 2:** Configuration of each plane.

Id	Position	Height	Width
1	100 mm	100 mm	700 mm
2	200 mm	200 mm	500 mm
3	300 mm	300 mm	300 mm
4	400 mm	400 mm	100 mm

**Table 3:** Real dataset.

Dataset	f [mm]	$f_{e}$ [fps]	Height [m]	GIFOV	Speed	Mode
Mametz 1	35	10	≈2.85	0.43 mm/px	≈2.6 mm/s	open loop
Mametz 2	12	10	2.85	1.29 mm/px	≈3.89 mm/s	open loop
Mametz 3	35	10	2.85	0.43 mm/px	2.15 mm/s	closed loop

## Data Availability

The data presented in this study are available on request from the corresponding author. The data are not publicly available due to privacy.
